# FIGO good clinical practice paper: management of the second stage of labor

**DOI:** 10.1002/ijgo.13552

**Published:** 2021-01-28

**Authors:** Alison Wright, Anwar H. Nassar, Gerry Visser, Diana Ramasauskaite, Gerhard Theron, for the FIGO Safe Motherhood and Newborn Health Committee

**Affiliations:** ^1^ Department of Obstetrics and Gynaecology Royal Free London Teaching Hospital London UK; ^2^ Department of Obstetrics and Gynecology American University of Beirut Medical Center Beirut Lebanon; ^3^ Department of Obstetrics University Medical Center Utrecht the Netherlands; ^4^ Center of Obstetrics and Gynaecology Vilnius University Faculty of Medicine Vilnius Lithuania; ^5^ Department of Obstetrics and Gynaecology Faculty of Medicine and Health Sciences Universiteit Stellenbosch Stellenbosch South Africa; ^6^ International Federation of Gynecology and Obstetrics (FIGO) London UK

**Keywords:** birth, cesarean, delivery, fistulae prevention, forceps, impacted fetal head, perinatal, physiological birth, quality, respectful maternity care, safety, second stage of labor, vacuum

## Abstract

This good clinical practice paper provides an overview of the current evidence around second stage care, highlighting the challenges and the importance of maintaining high‐quality, safe, and respectful care in all settings. It includes a series of recommendations based on best available evidence regarding length of second stage, judicious use of episiotomy, and the importance of competent attendants and adequate resource to facilitate all aspects of second stage management, from physiological birth to assisted vaginal delivery and cesarean at full dilatation. The second stage of labor is potentially the most dangerous time for the baby and can have significant consequences for the mother, including death or severe perineal trauma or fistula, especially where there are failures to recognize and repair. This paper sets out principles of care, including the vital role of skilled birth attendants and birth companions, and the importance of obstetricians and midwives working together effectively and speaking with one voice, whether to women or to policy makers. The optimization of high‐quality, safe, and personalized care in the second stage of labor for all women globally can only be achieved by appropriate attention to the training of birth attendants, midwives, and obstetricians. FIGO is committed to this aim alongside the WHO, ICM, and all FIGO’s 132 member societies.

## Abstract 2

This paper provides an overview of the current evidence in best practice for second stage care, highlighting the challenges of maintaining high‐quality, safe, and respectful care in all settings.

## 1 INTRODUCTION

Historically, international health policy and programming have tended to focus on the first stage of labor, including appropriate use of the partograph and identification of hypertension or sepsis, and on the third stage of labor, including active management and hemorrhage prevention. While these are important areas, this paper specifically addresses issues of the second stage of labor.

Besides the risks of the second stage of labor to the woman, there are data to suggest that the most dangerous time in an individual's life is at the time of one's own birth.^1^

This good clinical practice paper is an update of the FIGO Safe Motherhood and Newborn Health Committee's 2012 paper published in *International Journal of Gynecology & Obstetrics*.^2^ It is not intended to be a systematic review of the literature, rather a document to strengthen frameworks for service provision and to enable providers to improve care for women, in line with evidence‐based recommendations. In particular, it aims to highlight important global clinical and policy issues requiring attention relating to care in the second stage of labor.

According to the World Health Organization (WHO), the second stage of labor is defined as the period of time between full cervical dilatation and birth of the baby, during which the woman has an involuntary urge to bear down, as a result of expulsive uterine contractions.^3^

There is currently wide variation in the recommended length of the second stage and until this debate is resolved it is vital that global research into maternal and fetal outcomes related to second stage length is prioritized.

In most cases, during the second stage of labor, despite uteroplacental circulation being reduced, there is enough reserve to maintain oxygenation of the fetus until birth. However, there is potential for both the fetal and maternal condition to deteriorate rapidly during the second stage. Deterioration can occur in pregnancies with known risk factors, such as pre‐eclampsia or fetal growth restriction, but also, sometimes unpredictably, in apparently low‐risk pregnancies.^4^ Thus, antenatal risk assessment and progress in the first stage of labor, such as represented by a normal partogram, are not necessarily reliable predictors of normal outcomes and birth attendants must remain vigilant in all cases during the second stage.

Important potential complications arising in the second stage of labor are fetal hypoxia leading to birth asphyxia; failure of the presenting part to rotate or descend appropriately; and worsening or new maternal hypertension and pre‐eclampsia. Women with pre‐existing cardiac disease or severe anemia may be at risk of heart failure during the second stage owing to the additional circulatory demands of active pushing. Thus, high‐quality and safe care in the second stage of labor is essential to prevent stillbirth and newborn complications arising from asphyxia, as well as maternal mortality and morbidity from complications such as vesicovaginal fistula, anal sphincter injury, sepsis, hemorrhage, and worsening of hypertensive disease.^5^

During the second stage of labor, birth attendants should observe these 10 key principles of second stage care:

- Continuously provide accurate and honest information, support, and encouragement to the woman and her birth companion(s) and ensure she has the autonomy to make informed choices (unless she explicitly lacks the capacity to do so).
- Listen frequently (every 5 minutes, or after every contraction, whichever comes first) to the fetal heart, to detect fetal heart abnormalities. If deemed to be high‐risk, monitor continuously, using cardiotocograph, where available.
- Monitor the maternal pulse and blood pressure in every case, and more frequently where there is a pre‐existing problem of hypertension, pre‐eclampsia, anemia, or cardiac disease.
- Augment contractions with an intravenous oxytocin infusion during the second stage—when necessary, and only where safe to do so—if contractions have become infrequent, providing the fetal heart rate remains normal. This may avoid the need for assisted vaginal delivery (AVD) or transfer to a facility.
- Encourage active pushing if the cervix is fully dilated with less than 2/5 of the head palpable per abdomen and when the urge to bear down is present ^6^ (unless epidural in situ), with encouragement to adopt any position for pushing preferred by the woman except lying completely supine.
- Observe progressive descent and rotation of the presenting part. This includes observing progressive distension of the perineum and performing vaginal examination when required, especially where labor progress appears to be slow.
- Facilitate the birth with continuous communication with the woman and appropriate support of the perineum and control of the presenting part, to avoid significant perineal tears and obstetric anal sphincter injury (OASI).
- Perform an episiotomy only where a significant (more than second degree) tear or OASI is judged to be likely, or to expedite delivery in the presence of fetal distress (restrictive, rather than routine use).
- Use vacuum or forceps for AVD where indicated for suspected fetal compromise or non‐advancement of the fetal head, providing an appropriately skilled professional is available.
- Consider second stage cesarean birth if AVD is not safely feasible, usually because the head is at a high station. In this case the attending team should anticipate and prepare to avoid the potential associated complications. Suppression of contractions should be considered while preparing for cesarean delivery, especially where the decision to delivery time may be delayed.

## 2 WORKING WITH MIDWIVES

It is crucial that obstetricians work together with midwifery colleagues and that obstetricians and midwives speak with one voice when speaking to managers and policy makers. FIGO has a very close relationship with the International Confederation of Midwives (ICM), and the two organizations increasingly liaise on guidelines and policies. ICM supports, represents, and works to strengthen professional associations of midwives throughout the world. There are currently 143 Midwives’ Associations, representing 124 countries across every continent. ICM sets the international definition and scope of practice of a midwife (updated 2017) and defines the Essential Competencies for Midwifery Practice (updated 2019). In addition, ICM provides global standards for midwifery education and for midwifery regulation (both updated in 2011). These globally accepted definitions, competencies, and standards are used throughout the world by midwives and others working to strengthen midwifery in the maternal and newborn health sector. ICM’s Philosophy and Model of Midwifery Care describes pregnancy and childbirth as a usually normal physiological process that midwives strive to promote, protect, and support through care that is based on respect, compassion, and human rights.^7^, ^8^, ^9^

## 3 RESPECTFUL CARE

Every woman has the right to dignity, respect, and skilled care during pregnancy and childbirth but not every woman receives it.^10^

There is a recent, much welcomed interest in respectful care, and increasingly organizations are attempting to define and articulate what is meant by this. As with all aspects of maternity care, in accordance with a rights‐based and respectful care approach, the individual needs of the woman and her companion during the second stage of labor should be taken into consideration, therefore personalizing her care. Special consideration is required for culturally based birth preferences, especially where these are unusual or a minority within a healthcare setting. It is understood that lack of attention to respectful care by maternity care providers is a major barrier to the utilization of health facilities in many countries, as reflected in health surveys that show reasonable uptake of antenatal care but low rates of delivery in health facilities, and further work is required in this area.

Many facilities do not allow partners or companions to remain with women during labor. Whilst outdated hospital regulations may contribute, this may be partly due to the design of shared delivery rooms that lack privacy, such as partitions and curtains. During the COVID‐19 pandemic , these restrictions were also put in place to minimize spread of the infection.

## 4 SPECIFICS OF SECOND STAGE CARE

### 4.1 Birth attendants

This is the stage in labor where the contribution of a qualified and skilled birth attendant is probably the most critical in ensuring a safe outcome, besides technical knowledge.

While attending a delivery the timing of active pushing should be guided so that this is encouraged only when the cervix is fully dilated, when the presenting part has engaged in the pelvis, and the woman feels the urge to push (unless epidural is being used). The skilled attendant also has the role of encouraging the mother to adopt positions for active pushing that are comfortable and mechanically beneficial —for example, squatting or sitting. In many hospitals in low‐resource countries, lying supine in labor has been encouraged, a tendency which may be exacerbated by a lack of available cushions or the use of non‐flexible delivery beds where the upper part cannot be elevated.

Assuring safety and quality of second stage care, and provision of the 10 key elements above, requires the presence of a second birth attendant trained to assist^11^—e.g. to maintain auscultation of the fetal heart and support for the mother while the midwife or obstetrician puts on sterile gloves in preparation for the delivery. If complications occur the second birth attendant can then call for help and initiate emergency care, while not detracting from the continuous care provided to the mother by the skilled attendant. To achieve this, facilities providing maternity care need to structure their staff allocation and skill mix to recognize the extra care requirements in the second stage. Whilst this is challenging in settings where budgets or shortages of skilled staff are major constraints (as can also occur in high‐income settings), serious efforts to provide effective care at this critical stage can reduce the burden of emergency interventions for asphyxiated babies and mothers with preventable complications.

Special consideration is needed in delivery settings where only one skilled attendant is available, such as home births or small health centers. In these cases, birth planning needs to involve relatives, traditional birth attendants, or non‐clinical staff to assist in the role of the second birth attendant. Such assistants need to be briefed about their role and arrangements made for them to be accessible and present for the birth.

### 4.2 Duration of the second stage of labor

National and international organizations vary in their recommendations of what is an acceptable duration of the second stage, with ongoing international debate and some groups defending a longer duration. This demonstrates the need for more robust and targeted research into the duration of the passive and active second stage and maternal and fetal outcomes.

According to NICE guidelines (2014),

For a nulliparous woman:

- birth would be expected to take place within 3 hours of the start of the active second stage in most women.
- diagnose delay in the active second stage when it has lasted 2 hours and refer the woman to a healthcare professional trained to undertake an operative vaginal birth if birth is not imminent.

For a multiparous woman:

- birth would be expected to take place within 2 hours of the start of the active second stage in most women.
- diagnose delay in the active second stage when it has lasted 1 hour and refer the woman to a healthcare professional trained to undertake an operative vaginal birth if birth is not imminent.^12^

The American College of Obstetricians and Gynecologists (ACOG) Practice Bulletin No. 49 on Dystocia and Augmentation of Labor defines a prolonged second stage as more than 2 hours without or 3 hours with epidural analgesia in nulliparous women, and 1 hour without or 2 hours with epidural in multiparous women. This definition diagnoses 10% to 14% of nulliparous and 3% to 3.5% of multiparous women as having a prolonged second stage. Although in some ways modern obstetric population and practice have evolved with time, current labor management is still largely based on data established by Friedman in the 1950s.^13^

Controversy remains regarding the total length of second stage and as to whether pushing should be “delayed”. Some advocate for a longer second stage and have shown that approximately 78% of nulliparous women delivered vaginally even after 4 hours of pushing.^14^ A recent systematic review and meta‐analysis of the literature in *AJOG* evaluating the effect of delayed versus immediate pushing in women with neuroaxial analgesia showed that delayed pushing in the second stage does not affect the mode of delivery, although it reduces the time of active pushing at the expense of a longer second stage. This review also found that prolongation of labor was associated with a higher incidence of chorioamnionitis and low umbilical cord pH. Based on these findings, these authors conclude that delayed pushing cannot be routinely advocated for the management of the second stage.^15^

WHO, in its 2018 recommendations for intrapartum care for a positive childbirth experience, does not differentiate between the passive and active aspects of the second stage, nor whether an epidural is in situ or not. Women should be informed that the duration of the second stage varies from one woman to another. In first labors birth is usually completed within 3 hours, whereas in subsequent labors birth is usually completed within 2 hours.^16^ FIGO recommends that care providers follow these WHO recommendations.

### 4.3 Maternal and fetal monitoring during the second stage

The following observations are recommended in the second stage of labor. All observations should be recorded on the partograph to assess whether escalation, intervention, or transfer of care may be needed.

- Half‐hourly documentation of the frequency of contractions
- Hourly blood pressure
- Continued 4‐hourly temperature
- Frequency of passing urine
- Offer a vaginal examination hourly in the active second stage, or in response to the woman's wishes (after abdominal palpation)
- Perform intermittent auscultation of the fetal heart rate immediately after a contraction for at least 1 minute, at least every 5 minutes. Palpate the woman's pulse every 15 minutes to differentiate between the two heartbeats.^12^

The above are the minimum observations and assume a healthy (low‐risk) mother and fetus. If the woman's blood pressure is raised, or if fetal compromise is suspected, monitoring in the second stage should be more frequent.

Equipment in good working order and devices that simplify detection of the fetal heartbeat should be available. The birth attendant should have the skills to interpret the fetal heart rate and take appropriate action when needed. Whilst the traditional Pinard stethoscope may be adequate in very quiet labor rooms, it is often difficult to use reliably, especially in the second stage. Wide availability of handheld Doppler devices with battery backup and/or wind‐up recharging technology should be part of standard equipment provision for safe and high‐quality care in the second stage. Service planners and managers should therefore prioritize procurement and regular maintenance of such devices.

However, the recent Delphi consensus statement on fetal monitoring concluded that there is a gap between international recommendations and what is physically possible in many labor wards in low‐resource settings. Research on how to effectively implement the consensus on fetal assessment at admission and use of handheld Doppler during labor and delivery is crucial to support staff in achieving the best possible care in low‐resource settings.^17^

### 4.4 Position of the woman during the second stage of labor

Position changes during labor to enhance maternal comfort and promote optimal fetal positioning can be supported, so long as adopted positions allow appropriate maternal and fetal monitoring and treatments and are not contraindicated by maternal medical or obstetric complications.

Studies looking at optimizing the position of birth to achieve spontaneous vaginal delivery and to avoid AVD have tended to categorize women into those with epidural (generally low dose) and those without.

The use of any upright or lateral position in the second stage of labor, compared with supine or lithotomy positions, is associated with a reduction in AVDs in women not using epidural.^18^ A randomized trial included 3236 nulliparous women with a low‐dose epidural to determine whether being upright in the second stage of labor increases the chance of spontaneous vaginal birth compared with lying down. Significantly fewer spontaneous vaginal births occurred in women in the upright group compared with the lying down group (35.2% vs 41.1%; adjusted risk ratio 0.86, 95% confidence interval 0.78–0.94). This represents a 5.9% absolute increase in the chance of spontaneous vaginal birth in the lying down group.^19^ These results are interesting, especially given the apparently contrary data regarding position in women without an epidural, and clearly further work is required in this area.

Currently, the advice is that women should be supported to birth in a position of their own choice. All birthing facilities should therefore have adequate space, equipment, and skilled care providers to facilitate that.

### 4.5 Use of oxytocin during the second stage of labor

Oxytocin must be used with great caution, as if not appropriately used this drug is potentially dangerous for mother and fetus. Intramuscular oxytocin administration before delivery is absolutely contraindicated. Intravenous oxytocin can be used in the second stage of labor, with the aim of reducing the need for cesarean birth or AVD, if the contraction pattern is deemed inadequate and providing the fetal presentation, position, and heart rate have been confirmed to be normal. It is vital that a thorough and complete assessment of the situation and examination of the woman is performed to exclude cephalo‐pelvic disproportion or obstructed labor, prior to commencing oxytocin.

One review showed no statistically significant difference in AVD between women in spontaneous labor with epidural analgesia who were augmented with oxytocin and those who received placebo. The authors did comment that, owing to the limited number of women included in the studies, further research in the form of randomized controlled trials is required.^20^

Intravenous oxytocin should only be administered according to a facility protocol (describing indications, dose, and intravenous route) by a trained care provider, with contractions regularly palpated and monitored (should not be more than five in 10 minutes). Infusions based on counting drops in an intravenous giving set can result in inaccurate oxytocin dosing. If an infusion pump is not available, the resulting contraction frequency and strength should be observed extremely carefully to avoid hyperstimulation. Where one‐to‐one care is not feasible, these risks may outweigh the potential benefits and oxytocin should be used with extreme caution, if at all.

## 5 INTERVENTIONS TO PROMOTE PHYSIOLOGICAL VAGINAL BIRTH AND REDUCE THE NEED FOR AVD AND CESAREAN BIRTH

Various interventions have been shown to increase rates of spontaneous vaginal birth, including adequate hydration, different positions for birth, respectful care, and the presence of a birth companion.

Continuous support for women during childbirth by one‐to‐one birth attendants, especially when the care provider is not a member of staff, has been shown to reduce the need for AVD^21^ and data regarding access to a doula birth attendant suggest that increasing access to doula care for at‐risk women who desire intrapartum doula support may facilitate decreases in rates of ‘non‐indicated’ cesarean deliveries.^22^

## 6 ASSISTED VAGINAL DELIVERY (AVD)

If the aim for physiological vaginal birth is not achieved despite the above measures, or when abnormalities in the fetal heart rate are detected, the use of AVD (by vacuum extractor or forceps) may help shorten the second stage of labor and reduce the need for second stage cesarean birth and should be considered.^23^, ^24^

Before performing an AVD, a thorough assessment of the situation is necessary, and all prerequisites considered (Appendix A).^25^

AVD should only be attempted by healthcare providers who are trained and qualified to recognize the indications and who are skilled and equipped to perform the AVD safely. Appropriate training in technique and decision‐making is essential to ensure high‐quality and safe care for mother and baby; inadequate training has been shown to be a key contributor to adverse outcomes.^26^

In those countries where care providers other than obstetricians are required to perform AVD, appropriate training for them and supportive legislation should be in place.^27^ In the absence of formal legislation there should be a written document enabling the care provider to intervene and stating the circumstances under which this can be done. The aim of this policy is to enable providers to use their skills without fear of criticism arising from concerns about professional scope of practice, as well as ensuring safe care.

“Hands‐on” training in AVD as well as other aspects of second stage management is essential. Details of a recommended workshop are shown in Appendix B. It must be acknowledged, however, that isolated workshops are not in themselves adequate and must be followed up by ongoing support and supervision on the labor ward, especially in settings where AVD has become less commonly used. Hospitals and facilities need to provide appropriate obstetric instruments and ensure that care providers are appropriately trained and competent to use them.

Regarding the choice of instrument for AVD, this is dependent on a balance of clinical circumstance and practitioner experience.^12^ A Cochrane review has included evidence from 10 trials evaluating the relative merits of vacuum versus forceps delivery.^28^ Overall, vacuum delivery appears to be associated with reduced maternal trauma compared with forceps, whilst the failure rate appears to be reduced with forceps. However, when looking at outcomes following use of vacuum and forceps it should be noted that there is a paucity of randomized trials in this area and therefore comparative data should be interpreted with caution. Whichever instrument is used, AVD should not be about force, but about flexion and realignment. The importance of identifying the flexion point is crucial. This is certainly the key to any successful vacuum delivery and is often overlooked, especially by those who receive little to no training. Handheld vacuum devices such as the Kiwi Omni Cup have become popular as these are easy to use, with the attendant being able to control the suction. Cheaper, reusable options are currently being developed. Simple, risk‐based information for women and care providers regarding vacuum and forceps is summarized in the consent advice from the Royal College of Obstetricians and Gynaecologists.^29^

Currently undergoing testing by WHO and global partners is a new low‐cost device for AVD, the Odon device. This device is applied using a simple inserter and although the mechanism is not yet completely understood, it appears to work on the principle of flexion of the head, facilitating delivery. The Odon has been designed for ease of use with minimal training in low‐resource settings. WHO is implementing a three‐phased study protocol, but until the device has been fully evaluated it cannot be recommended for routine use.^30^

According to new evidence from 2019,^31^ benefit of a single dose of prophylactic antibiotic after AVD has been shown, although this has not yet been universally included in guidelines.

## 7 VAGINAL BREECH

Vaginal breech delivery is undertaken when a woman opts for this, or where the balance of risk is considered to favor it over cesarean delivery, which may occur where access to cesarean delivery is limited. All skilled attendants need to be familiar with the diagnosis of breech presentation in labor and with maneuvers for vaginal breech delivery using simulation, because in many settings it is increasingly unlikely that birth attendants will undertake enough vaginal breech deliveries to maintain competency without simulation training.

## 8 PAIN RELIEF DURING THE SECOND STAGE OF LABOR

Where there is a delay in the second stage of labor, or if the woman is excessively distressed, support and sensitive encouragement and the woman's potential requirement for analgesia/anesthesia are particularly important considerations.^12^

Pain relief options should be discussed with the woman prior to the onset of labor and offered according to her wishes, facility protocols, and available resources. Women should be encouraged to prepare a personalized care and support plan (PSCP), including pain relief preferences, in conjunction with their care provider. The need for pain relief is highly variable between individuals and should be individually assessed. Care providers should not base assumptions of “coping” on visible pain behavior. There is debate regarding epidural analgesia and its effect on the second stage of labor. The PEOPLE study showed that the inability to sustain optimal epidural analgesia is associated with an increased risk of adverse second‐stage obstetric outcomes.^32^

Epidural or regional analgesia is not consistently available, especially in some low‐ and middle‐income countries, and availability should be considered when offering choice.^33^

Local anesthesia should be used for perineal infiltration prior to performing an episiotomy, and the practice of cutting an incision without anesthesia should now be obsolete. For AVD, a pudendal block may be used.

## 9 EPISIOTOMY

An episiotomy is an incision made in the perineum, either when a significant tear is judged to be likely or for a breech presentation, or to decrease the length of the second stage of labor in cases of fetal distress. There are some conflicting data regarding a policy of restricted episiotomy (episiotomy only when necessary) versus a policy of routine episiotomy regarding maternal and fetal outcomes. FIGO is clear in its support of restrictive rather than routine use of episiotomy.^34^

When performing episiotomy, mediolateral episiotomy is generally recommended,^35^ especially for AVD, where it appears to protect against OASI. A mediolateral episiotomy should be performed at 60 degrees.^35^ A large observational study from the Netherlands of 28 732 assisted vaginal births concluded that mediolateral episiotomy is protective against OASI in both vacuum extraction (9.4% vs 1.4%) and forceps birth (22.7% vs 2.6%).^36^

An episiotomy should always be performed under adequate analgesia, whether anesthesia is already in place for labor, such as epidural, or by administering a local infiltration.

## 10 OBSTETRIC ANAL SPHINCTER INJURY (OASI)/SEVERE PERINEAL TRAUMA

Every attempt should be made to ensure that OASI or third‐ and fourth‐degree tears (sometimes referred to as “severe perineal trauma”) do not occur, by supporting the perineum and controlling the presenting part. When they do occur, all birth attendants should be trained in recognition and escalation for appropriate repair.^37^ Various interventions have been shown to prevent third‐ and fourth‐degree tears, such as the use of a warm compress on the perineum during the second stage.^38^

## 11 SECOND STAGE CESAREAN BIRTH

Simulation training in the specific challenges of second stage cesarean delivery should be provided wherever cesarean services exist. Training should include decision‐making regarding whether AVD or cesarean delivery would be the safer option, and skills and drills in how to deliver an impacted fetal head at a cesarean birth. In settings where it is available, the fetal pillow can be used to assist with disimpacting the fetal head.^39^

## 12 FEMALE GENITAL MUTILATION (FGM)

FIGO and WHO are absolutely opposed to all forms of FGM and are absolutely opposed to healthcare providers performing FGM (medicalization of FGM). If presenting in labor, especially in the second stage, presence of grade 3 FGM with obstruction of the vaginal introitus following infibulation requires attending staff to be appropriately trained in deinfibulation. Best practice consists of antenatal identification of women with FGM and the offer of deinfibulation before the onset of labor, supported by appropriate counseling, to facilitate safety in the second stage.^40^ If not performed antenatally, when a woman presents in labor, deinfibulation should be undertaken when the tissues are stretched as the fetal head descends. Deinfibulation should be performed before evaluating the need for episiotomy, which may therefore not be required.

## 13 FUNDAL PRESSURE

There is currently insufficient evidence to draw conclusions on the beneficial or harmful effects of fundal pressure and therefore it is generally accepted that more research is required in this area.^41^

Although fundal pressure is still used in some settings, given its safety is as yet unproven, WHO and FIGO do not recommend its use.^42^

## 14 IMPLICATIONS FOR HEALTH SYSTEMS IN LOW‐ AND MIDDLE‐INCOME COUNTRIES

Clinical interventions (AVD, cesarean) during the second stage of labor should only be offered or advised where labor is not progressing normally and/or where there are concerns about mother and baby and, crucially, should only be initiated when appropriately trained staff and equipment are in place.^12^

Probably the biggest problems currently affecting second stage care in many low‐ and middle‐income countries are inadequate numbers of skilled healthcare attendants and non‐respectful maternity care (which may well be related). There are concerns that birth attendants do not have the means or time to monitor the fetus, combined with a lack of access and knowledge regarding AVDs and inadequate facilities and experience to perform a safe second stage cesarean delivery.

Close attention to the maternal and the fetal condition during the second stage is essential to provide the necessary clinical reassurance that no interventions are necessary, or otherwise. If the conditions deviate from normal, options for immediate intervention or referral (depending on the care setting) should be defined clearly in local protocols and guidelines to allow timely access to emergency obstetric and neonatal care.

Depending on the level of the healthcare system where the care is provided, the skilled attendant and the assistant should have access to equipment for AVD and neonatal resuscitation and should have the appropriate skills to use that equipment.

In settings where only one skilled attendant is available, briefing of relatives or non‐clinical staff about their roles is required.

In many settings, there are challenges with consistent provision of aspects of second stage care at various levels of the health system. According to Service Provision Assessments in several African countries, AVD was notably lacking.^43^ This means that expediting delivery of the baby in the second stage would not be possible, even when an abnormal fetal heart rate is detected.

Policy makers need to develop and implement sustainable plans for ensuring that the necessary human resources, skills, and equipment are in place in a structured manner at every level of the health system. Transfer to another facility during the second stage of labor is potentially dangerous and may be associated with poor outcomes because of the additional delay. Thus, every effort should be made to provide the AVD component of Basic Emergency Obstetric Care, so that safe delivery can be performed at a health center, without the need for transfer.

## 15 SECOND STAGE OF LABOR IN THE COVID‐19 PANDEMIC

There is no evidence that the second stage of labor should be managed differently during the COVID‐19 pandemic, unless the woman is clinically deteriorating. Appropriate PPE should be worn by attendants of labor, including in the second stage, in accordance with local guidelines ^44^ and there may need to be some restriction on the number of attending companions.

## 16 RECOMMENDATIONS

- Women should be informed that the duration of the second stage varies from one woman to another. In first labors, birth is usually completed within 3 hours whereas in subsequent labors, birth is usually completed within 2 hours (in accordance with WHO recommendations)
- Birthing facilities must offer every woman respect and privacy where possible and allow her to be accompanied by her choice of a supportive person (husband, friend, mother, relative), unless there are infection control measures in place (e.g., during the COVID‐19 pandemic)
- Psychosocial support, education, communication, choice of position, and pharmacological methods which are appropriately used during the first stage are also useful in relieving pain and distress in the second stage of labor
- There should be at least two people assisting at every birth, whether it is another health professional, family member, second birth attendant, or village health worker. Arrangements for having another person besides the primary skilled attendant should be planned for during the antenatal period
- Monitoring of the fetal heart must be continued during the second stage to allow early detection of fetal compromise
- Health facilities and skilled attendants should be provided with handheld battery‐powered or handheld Dopplers for fetal heart auscultation after every contraction, particularly in the active phase of the second stage. These should be added to lists of essential commodities
- Routine episiotomy should not be practiced. Restrictive (judicious) use of episiotomy is recommended
- Women without an epidural should not be encouraged to push until they feel an urge to push, unless the recommended second stage duration is exceeded
- Local anesthetic should be given for any episiotomy, perineal tear repair, or AVD where regional analgesia is either not available,not appropriate, or not requested
- The lack of access to AVD appears to be a major deficit in obstetric care in many facilities globally and needs to be urgently addressed. Skills necessary for safe AVD, safe second stage, cesarean birth and related decision‐making must be integrated into pre‐service and in‐service education for all skilled attendants
- Provision of critical skills for second stage management needs to be supported by policies as well as simulation training and linkage with a functioning referral system.

## 17 RECOMMENDED RESEARCH FOR SECOND STAGE MANAGEMENT

1. What are the short‐, medium‐, and long‐term outcomes in relation to the duration of the second stage for women and babies? (Longer‐term may include prolapse and incontinence for the woman and neurodevelopmental parameters in the child.)
2. What is the role of targeted or routine use of abdominal or perineal ultrasound (where available) for assessment of the station, flexion, and descent of the fetal head in the second stage of labor?
3. Future research into effects of fundal pressure should describe in detail how the pressure was applied and consider safety of the unborn fetus, perineal outcomes, longer‐term maternal and infant outcomes, and maternal satisfaction.
4. Is there an association between vacuum delivery and mother‐to‐child transmission of HIV? Since a randomized controlled trial would not be ethical or feasible, a retrospective case–control study or observational study would be the preferred study design.
5. What maneuvers and devices can assist fetal head impaction at second stage cesarean birth?
6. What is the evidence base around lack of respectful care acting as a deterrent to women accessing care in a facility?

## MEMBERS OF THE FIGO SAFE MOTHERHOOD AND NEWBORN HEALTH COMMITTEE, 2018–2021

Anwar Nassar (chair), Gerard H. A. Visser (past chair), Eytan R. Barnea, Maria Fernanda Escobar, Yoon Ha Kim, Wanda Kay Nicholson, Rodolfo Pacagnella, Diana Ramasauskaite, Gerhard Theron, Alison Wright.

## Prerequisites for AVD

### 1

Adapted with permission from Table 3, Murphy, DJ, Strachan, BK, Bahl, R, on behalf of the Royal College of Obstetricians & Gynaecologists. ‘Assisted Vaginal Birth’. BJOG 2020; 127: e70–e112.

Full abdominal and vaginal examination.

- Head is ≤1/5 palpable per abdomen (in most cases not palpable)
- Cervix is fully dilated, and the membranes ruptured
- Station at level of ischial spines or below
- Position of the fetal head has been determined
- Caput and moulding is no more than moderate (or +2)
- Pelvis is deemed adequate
- Preparation of mother
- Clear explanation given and informed consent taken and documented in women's case notes
- Trust established and full cooperation sought and agreed with woman
- Appropriate analgesia is in place: for mid‐pelvic or rotational birth, this will usually be a regional block; a pudendal block may be acceptable depending on urgency; and a perineal block may be enough for low or outlet birth.
- Maternal bladder has been emptied
- Indwelling catheter has been removed or balloon deflated
- Aseptic technique
- Preparation of staff
- Operator has the knowledge, experience and skill necessary
- Adequate facilities are available (equipment, bed, lighting) and access to an operating theatre.
- Backup plan: for mid‐pelvic births, theatre facilities should be available to allow a caesarean birth to be performed without delay; a senior obstetrician should be present if an inexperienced obstetrician is conducting the birth
- Anticipation of complications that may arise (e.g. shoulder dystocia, perineal trauma, postpartum haemorrhage).
- Personnel present who are trained in neonatal resuscitation

## QSSS RCOG/FIGO workshop

### 1

Quality and Safety in the Second Stage (QSSS)

This workshop is for doctors working in maternity services and any other professionals competent to perform cesarean delivery. It is part of the ongoing RCOG QSSS training package which promotes not only the appropriate, safe, and successful use of operative vaginal delivery (vacuum and forceps) but also focuses on clinical decision making in the second stage of labor to enable high‐quality and safe care for women at the end of their labor.

The workshop will also include other quality improvement issues in second stage of labor management such as the prevention of obstetric anal injuries and techniques to minimize the complications of second stage cesarean section. Our experienced international faculty come from high‐ and low‐resource environments and we will consider the impact of resource when delivering the modules. The workshop style will be small group teaching together with practical, “hands on” methods and a small proportion of lecture time.

This workshop has the potential to reduce unnecessary cesarean sections and, where they are performed, to minimize complications, saving women's lives. Other benefits may be to reduce rates of obstetric anal sphincter injuries and increase the accurate identification and grading of the injuries, as well as reducing rates of iatrogenic and obstructive fistula.

There is also a professional and advocacy aspect to QSSS. We believe that there is a role for the RCOG and FIGO in clearly recommending that doctors in training should gain skills in assisted vaginal delivery, rather than seeing cesarean section as the “easy option” when there are problems in the second stage of labor.

## References

[1] Walker KF , Cohen AL , Walker SH , Allen KM , Baines DL , Thornton JG . The dangers of the day of birth. BJOG. 2014;121:715‐719. 10.1111/1471-0528.12544 24521517

[2] FIGO Safe Motherhood and Newborn Health (SMNH) Committee . Management of the second stage of labor. Int J Gynaecol Obstet. 2012;119(2):111‐116. 22980427 10.1016/j.ijgo.2012.08.002

[3] WHO recommendation on definition and duration of the second stage of labour. 17 February 2018.

[4] Janni W , Schiessl B , Peschers U , et al. The prognostic impact of a prolonged second stage of labor on maternal and fetal outcome. Acta Obstet Gynecol Scand. 2002;81(3):214‐221. 11966477 10.1034/j.1600-0412.2002.810305.x

[5] Albers LL , Sedler KD , Bedrick EJ , Teaf D , Peralta P . Factors related to genital tract trauma in normal spontaneous vaginal births. Birth. 2006;33(2):94‐100. 16732773 10.1111/j.0730-7659.2006.00085.x

[6] Intrapartum care ‐ An advanced‐midwife’s guide to labour and delivery. https://www.glowm.com/pdf/2011–Intrapartum‐Care‐Free‐Online‐Edition‐EBWHealthCare‐CC%20BY%20NC%20ND.pdf. Published: February 16, 2011. Accessed December 16, 2020.

[7] International definition of the midwife. https://www.internationalmidwives.org/assets/files/definitions‐files/2018/06/eng‐definition_of_the_midwife‐2017.pdf. Published: 2017. Accessed December 16, 2020.

[8] Essential competencies for midwifery practice 2019 update. https://www.internationalmidwives.org/assets/files/general‐files/2019/10/icm‐competencies‐en‐print‐october‐2019_final_18‐oct‐5db05248843e8.pdf. Published: October 2019. Accessed December 16, 2020.

[9] International Confederation of Midwives . Global Standards for Midwifery Education (2010). Amended 2013. https://www.internationalmidwives.org/assets/files/general‐files/2018/04/icm‐standards‐guidelines_ammended2013.pdf. Accessed December 16, 2020. 10.1016/j.midw.2011.04.001 21550149

[10] Achieving respectful care for women and babies. Lancet. 2015;385(9976):1366. 10.1016/S0140-6736(15)60701-2 25890402

[11] World Health Organization . Making Pregnancy Safer: The Critical Role of the Skilled Attendant. A Joint Statement by WHO, ICM and FIGO. Geneva: WHO; 2004 http://whqlibdoc.who.int/publications/2004/9241591692.pdf

[12] NICE guidance Intrapartum care for healthy women and babies (CG190), 2014. 10.1136/bmj.g6886 PMC4707719 25472418

[13] Cheng YW , Caughey AB . Defining and managing normal and abnormal second stage of labor. Obstet Gynecol Clin North Am. 2017;44(4):547‐566. 29078938 10.1016/j.ogc.2017.08.009

[14] Grobman WA , Bailit J , Lai Y , et al.; Eunice Kennedy Shriver National Institute of Child Health and Human Development (NICHD) Maternal‐Fetal Medicine Units (MFMU) Network . Association of the duration of active pushing with obstetric outcomes. Obstet Gynecol. 2016;127(4):667‐673. 26959213 10.1097/AOG.0000000000001354 PMC4805446

[15] Di Mascio D , Saccone G , Bellussi F , et al. Delayed versus immediate pushing in the second stage of labor in women with neuraxial analgesia: a systematic review and meta‐analysis of randomized controlled trials. Am J Obstet Gynecol. 2020;223(2):189‐203. 32067972 10.1016/j.ajog.2020.02.002

[16] WHO recommendations: intrapartum care for a positive childbirth experience, 9 July 2018. 30070803

[17] Housseine N , Punt MC , Browne JL , et al. Delphi consensus statement on intrapartum fetal monitoring in low‐resource settings. Int J Gynaecol Obstet. 2019;146(1):8‐16. 30582153 10.1002/ijgo.12724 PMC7379246

[18] Gupta JK , Sood A , Hofmeyr GJ , Vogel JP . Position in the second stage of labour for women without epidural analgesia. Cochrane Database Syst Rev. 2017;5:CD002006. 28539008 10.1002/14651858.CD002006.pub4 PMC6484432

[19] Epidural and Position Trial Collaborative Group . Upright versus lying down position in second stage of labour in nulliparous women with low dose epidural: BUMPES randomised controlled trial. BMJ. 2017;359:j4471. 29046273 10.1136/bmj.j4471 PMC5646262

[20] Costley PL , East CE . Oxytocin augmentation of labour in women with epidural analgesia for reducing operative deliveries. Cochrane Database Syst Rev. 2013;(7):CD009241. 23846738 10.1002/14651858.CD009241.pub3 PMC7133539

[21] Hodnett ED , Gates S , Hofmeyr GJ , Sakala C , Weston J . Continuous support for women during childbirth. Cochrane Database Syst Rev. 2011;(2):CD003766. 21328263 10.1002/14651858.CD003766.pub3

[22] Kozhimannil KB , Attanasio LB , Jou J , Joarnt LK , Johnson PJ , Gjerdingen DK . Potential benefits of increased access to doula support during childbirth. Am J Manag Care. 2014;20(8):e340‐e352. 25295797 PMC5538578

[23] Bailey PE . The disappearing art of instrumental delivery: time to reverse the trend. Int J Gynecol Obstet. 2005;91(1):89‐96. 10.1016/j.ijgo.2005.05.016 16109417

[24] Spencer C , Murphy D , Bewley S . Caesarean section in the second stage of labour. BMJ. 2006;333:613‐614. 16990297 10.1136/bmj.38971.466979.DE PMC1570839

[25] Murphy DJ , Strachan BK , Bahl R ;on behalf of the Royal College of Obstetricians Gynaecologists . Assisted Vaginal Birth (Green top guideline no. 26). BJOG. 2020;127:e70‐e112. 32346983 10.1111/1471-0528.16092

[26] Johnstone T . Minimizing risk: obstetric skills training. Clinical Risk. 2003;9:99‐102.

[27] World Health Organization . Task Shifting. Global Recommendations and Guidelines. Geneva: WHO; 2008 http://www.who.int/healthsystems/TTR‐TaskShifting.pdf

[28] Johanson RB , Menon BK . Vacuum extraction versus forceps for assisted vaginal delivery. Cochrane Database Syst Rev. 2000;2:CD000224. 10.1002/14651858.CD000224 10796182

[29] Royal College of Obstetricians and Gynaecologists . Operative Vaginal Delivery. Consent Advice No. 11. London: RCOG; 2010.

[30] Schvartzman JA , Carroli D , Di Renzo GC , et al. 0607 The ODON device. A new simple instrument for assisted vaginal delivery. Int J Gynecol Obstet. 2012;119:S475‐S476.

[31] Knight M , Chiocchia V , Partlett C , et al.; on behalf of the ANODE collaborative group* . Prophylactic antibiotics in the prevention of infection after operative vaginal delivery (ANODE): a multicentre randomised controlled trial. Lancet. 2019;393:2395‐2403. 31097213 10.1016/S0140-6736(19)30773-1 PMC6584562

[32] Abenhaim HA , Fraser WD . Impact of pain level on second‐stage delivery outcomes among women with epidural analgesia: results from the PEOPLE study. Am J Obstet Gynecol. 2008;199(5):500.e1‐500.e6. 18565489 10.1016/j.ajog.2008.04.052

[33] Dobson M . Opinion: labour analgesia in the developing world; why not. Rev Pain. 2011;5(3):2‐3. 10.1177/204946371100500302 PMC4590078 26526585

[34] Nassar AH , Visser GHA , Ayres‐de‐Campos D , Rane A , Gupta S ; FIGO Safe Motherhood and Newborn Health Committee . FIGO Statement: Restrictive use rather than routine use of episiotomy. Int J Gynaecol Obstet. 2019;146(1):17‐19. 31058312 10.1002/ijgo.12843

[35] Kapoor DS , Thakar R , Sultan AH . Obstetric anal sphincter injuries: review of anatomical factors and modifiable second stage interventions. Int Urogynecol J. 2015;26:1725‐1734. 26044511 10.1007/s00192-015-2747-0

[36] de Leeuw JW , de Wit C , Kuijken JP , Bruinse HW . Mediolateral episiotomy reduces the risk for anal sphincter injury during operative vaginal delivery. BJOG. 2008;115(1):104‐108. 17999693 10.1111/j.1471-0528.2007.01554.x

[37] Diko S , Guiahi M , Nacht A , et al. Prevention and management of severe obstetric anal sphincter injuries (OASIs): a National Survey of Nurse Midwives. Int Urogynecol J. 2020;31(3):591‐604. 30877353 10.1007/s00192-019-03897-x

[38] Aasheim V , Nilsen AB , Lukasse M , Reinar LM . Perineal techniques during the second stage of labour for reducing perineal trauma. Cochrane Database Syst Rev. 2011;(12):CD006672. 22161407 10.1002/14651858.CD006672.pub2

[39] Insertion of a balloon device to disimpact an engaged fetal head before an emergency caesarean section Interventional procedures guidance. Published: March 27, 2015. https://www.nice.org.uk/guidance/IPG515. Accessed December 16, 2020.

[40] RCOG 53 – Female Genital Mutilation and its Management (Green‐top Guideline No. 53) Published: July 10, 2015. https://www.rcog.org.uk/en/guidelines‐research‐services/guidelines/gtg53/. Accessed December 16, 2020.

[41] Hofmeyr GJ , Vogel JP , Cuthbert A , Singata M . Fundal pressure during the second stage of labour. Cochrane Database Syst Rev. 2017;3(3):CD006067. 28267223 10.1002/14651858.CD006067.pub3 PMC6464399

[42] WHO Reproductive Health Library . WHO Recommendation on Fundal Pressure to Facilitate Childbirth (February 2018). The WHO Reproductive Health Library. Geneva: World Health Organization.

[43] WHO, Unfpa, UNICEF, AMDD . Monitoring Emergency Obstetric Care: A Handbook. Geneva: WHO; 2009 http://whqlibdoc.who.int/publications/2009/9789241547734_eng.pdf.

[44] Poon LC , Yang H , Dumont S , et al. ISUOG Interim Guidance on coronavirus disease 2019 (COVID‐19) during pregnancy and puerperium: information for healthcare professionals ‐ an update. Ultrasound Obstet Gynecol. 2020;55(6):848‐862. 32356590 10.1002/uog.22061 PMC7267438

